# Multilevel Multimodal Physical Unclonable Functions by Laser Writing of Silicon Carbide Color Centers

**DOI:** 10.3390/mi16030329

**Published:** 2025-03-12

**Authors:** Yuxing Ma, Yue Qin, Hao Guo, Ye Tian, Lishuang Liu

**Affiliations:** State Key Laboratory of Extreme Environment Optoelectronic Dynamic Testing Technology and Instrument, North University of China, Taiyuan 030051, China; mayuxing96596@163.com (Y.M.); qytgyx@163.com (Y.Q.); tianye080t@163.com (Y.T.); lls@nuc.edu.cn (L.L.)

**Keywords:** PUF, multilevel multimodal, offsets, high encoding capability, integration, anti-counterfeiting

## Abstract

Information security serves as the cornerstone for ensuring the stable development of today’s highly digitized era. As cryptographic primitives with high security and robust encryption capabilities, physical unclonable functions (PUFs) are recognized as one of the critical solutions to address information leakage issues. However, the encoding of PUFs often relies on the inherent properties of materials, which limits the potential for further enhancement of their encoding capacity (EC). In this study, we introduce a four-level encoding scheme by leveraging the stochastic characteristics of free radical chemical reactions and energy deposition in the fabrication process of silicon carbide (SiC) color centers. A multilevel multimodal PUF (MMPUF) encoding strategy (ES) for flexible substrates with high EC, low cost, and simple and fast readout was constructed. The spatially random distribution of SiC and silicon vacancy (V_si_) color-center concentrations as well as the offsets of the laser pyrolysis position along the *X*- and *Y*-axes are four independent physical properties that ensure the encoding performance of the PUF, achieving a high encoding capacity of 2^4×10×10^ and secure, stable, and unclonable encoding. Furthermore, the integration of the PUF tags with the products through a doping manufacturing process, rather than simple attachment, enhances the security and practicality of the anti-counterfeiting system. The proposed encoding hierarchy based on the offsets provides a novel encoding solution for improving PUF EC.

## 1. Introduction

In the context of accelerating digitalization and globalization, information technology has profoundly permeated various fields, rendering information security a critical element for ensuring social stability and economic development [1,2,3]. As the value of data increases, security issues such as data leakage, identity fraud, and counterfeit products have become rampant, posing serious threats to personal privacy, commercial confidentiality, and even national security [4,5,6,7,8]. There is an urgent need to develop robust anti-counterfeiting strategies to combat the rising prevalence of counterfeit and substandard products. Recently, physical unclonable functions (PUFs) have emerged as a promising approach, garnering widespread interest [9].

The concept of physical one-way function (POF) was first introduced by Pappu et al. [10], which established the core principles for the PUF technology. POF is a security mechanism rooted in the randomness of physical properties, designed to generate unique and unclonable identifiers. PUF is a unique “fingerprint”-like identifier created by the random uncertainty introduced during the manufacturing process in non-ideal environments [11,12,13,14,15,16]. When a specific input is applied to a PUF tag, a uniquely corresponding result is produced [17]. Typically, the input applied to the PUF is referred to as a “challenge”, and the corresponding output is termed as a “response”. A pair of challenge and response is collectively known as a challenge response pair (CRP) [18].

In recent years, the development of various PUFs based on optical scattering [9], circuit characteristics [19], and biological cell colonization patterns [2] has provided an effective anti-counterfeiting method for information security. However, the development of deep learning technology has put forward higher requirements for the coding ability of PUFs. Currently, researchers have developed various multilevel PUF architectures to meet the higher security and encryption demands in complex scenarios [20,21,22]. These multilevel PUFs significantly increase the diversity of responses and the system’s resistance to attack by introducing more layers of encoding sources [23,24,25,26,27,28]. However, the encoding capacity of PUFs is often constrained by the inherent properties of the materials themselves, resulting in limited encoding characteristics [29]. Implementing multilevel PUF encoding often comes with increased design complexity, including a reliance on advanced manufacturing processes and the need for additional external devices to verify the behavior of CRPs. This not only escalates the manufacturing costs, but may also impose higher demands on their deployment and maintenance in practical applications.

To address these challenges, this study presents a high-capacity and low-cost multilevel multimodal PUF (MMPUF) encoding strategy (ES) that overcomes the limitations of existing encoding methods. By leveraging the unique photoluminescent properties of silicon carbide (SiC) and the characteristics of laser heat deposition during the manufacturing process, we propose a four-level PUF anti-counterfeiting strategy that utilizes the spatial random distribution of SiC and silicon vacancy (V_si_) centers as well as the positional offsets of laser ablation relative to a reference position along the *X*- and *Y*-axes. Compared with PUFs based on a single physical property, the MMPUF exponentially increases the traditional encoding capacity (EC) from E^F^ (E: the number of responses per pixel; F: the number of pixels) to E^4F^. The multimodal PUF fabricated via chemical vapor deposition (CVD) growth [7] involves complex fabrication processes, leading to higher manufacturing costs. Similarly, multilevel PUFs leveraging the optically detected magnetic resonance (ODMR) of the diamond spin structure [30] require additional validation devices, further increasing system complexity. In contrast, the MMPUF tag, enabled by laser direct writing (LDW), provides a highly flexible and cost-effective fabrication approach, significantly reducing the manufacturing expenses and minimizing dependence on complex external equipment [31,32]. Moreover, the PUF tag achieves product integration through doping manufacturing, enhancing concealment and tamper resistance. Additionally, the introduction of a flexible substrate broadens the applicability of the PUF tag, enabling deployment across diverse scenarios. Furthermore, the proposed offset-based encoding method establishes a novel paradigm for enhancing the EC of PUFs.

## 2. Materials and Methods

### 2.1. Manufacturing of MMPUF Labels

Using polydimethylsiloxane (PDMS) as the substrate, polyvinyl chloride (PVC) powder was incorporated at a weight ratio of 10:1. The mixed solution was thoroughly homogenized using an ultrasonic homogenizer (Model FB 505, Fisher Scientific, Waltham, MA, USA). Subsequently, the mixture was poured into a Petri dish and spin-coated using a spin coater. Afterward, it was transferred to a heating platform for annealing and curing at 80 °C for 40 min, resulting in a flexible thin film in sheet form. The film was then subjected to ablation using a continuous laser with a uniform low-energy distribution (532 nm green laser), which produces PCS and PCS-C. A self-designed gas chamber and connected tubing system were employed to create an inert gas environment. Finally, within this inert gas environment, the precursors were processed using a focused Gaussian laser, leading to the formation of silicon carbide nanocrystals and their spin ensembles. Consequently, the MMPUF labels were successfully manufactured.

### 2.2. Information Acquisition of MMPUF Labels

Optical images: The MMPUF tags were observed using an optical microscope (BX53M, OLYMPUS, Tokyo, Japan) at a magnification of 200×. The optical images were acquired using the built-in CCD camera (DP73) of the microscope, controlled by the associated software OLYSIM 2015 on a host computer.

Raman signals: A continuous-wave 532 nm green solid-state laser with a power of 200 mW was used as the excitation source for the Raman spectroscopy. The MMPUF tags were analyzed using a Raman spectrometer to obtain Raman mapping information, which was then processed numerically.

PL signals: A continuous-wave 730 nm laser with a power of 50 mW served as the excitation source. The light passed through a lens and then through an FELH0850 bandpass filter. The PL images of the MMPUF tags were captured using an infrared camera (Bobcat-640-GigE-8605, Xenics, Leuven, Belgium), with data acquisition controlled by the Xeneth 2.6 software on the host computer. The pixel gray values of the PL images were extracted and summed using MATLAB R2023a to obtain the numerical value of the PL signal.

*X*- and *Y*-axis offsets: By processing the collected optical images of the MMPUF tags, the coordinates of the actual pyrolysis positions and reference positions were marked in the XY coordinate system. The differences were calculated to determine the offsets along the *X*- and *Y*-axes.

### 2.3. Encoding of MMPUF Labels

The Raman signal intensity, PL intensity, and *X*- and *Y*-axis offsets were processed numerically using MATLAB R2023a, producing thermal maps for each level of encoding. Based on the design principles of the maximum entropy method, optimal thresholds were determined for binarization, resulting in 0 and 1 encoded images for each encoding level.

### 2.4. Calculation of Encoding Performance of MMPUF

Reproducibility and uniqueness were calculated by introducing the intra-HD and inter-HD. A three-dimensional matrix was established using MATLAB R2023a. The intra-HD was computed by analyzing the responses of the same PUF’s encoding levels over different times and mechanical wear, calculated row by row and element by element. The inter-HD was determined by calculating the responses of different PUFs at each encoding level, conducted row by row and element by element. To measure the spatial autocorrelation of the MMPUF tags, the spatial autocorrelation coefficient was calculated. Finally, Gaussian fitting was performed on the resulting statistical graphs.

## 3. Results and Discussion

### 3.1. Construction of MMPUF

**Figure 1:**
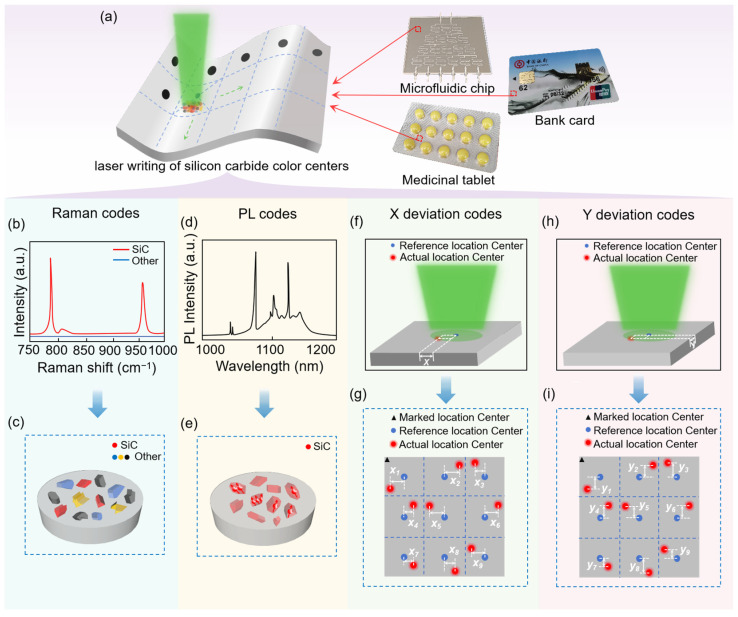
Construction of the MMPUF ES. (**a**) LDW of flexible substrates to prepare SiC color centers and their applications. (**b**) Raman spectroscopy. (**c**) Concentration distribution of the manufactured products. (**d**) Photoluminescence spectrum. (**e**) Distribution of color center concentration. The actual pyrolysis positions during the manufacturing process shifted relative to the reference position along the *X*-axis (**f**) and *Y*-axis (**h**). The offsets at each processing position along the *X*-axis (**g**) and *Y*-axis (**i**).

In order to improve the EC of PUFs and reduce their complexity and cost, a MMPUF structure was constructed in this study by taking full advantage of the multiple stochastic properties of the LDW induced pyrolysis process. This pyrolysis process, driven by laser direct writing, exhibits significant randomness in chemical reactions, laser distribution, and thermal deposition. By leveraging the spatially random distribution of SiC and its color centers as well as the positional offsets of the laser processing relative to a reference position along the *X*- and *Y*-axes, we designed a four-level anti-counterfeiting strategy for PUFs. As shown in Figure 1a, the products generated from the LDW on flexible substrates exhibited a random spatial distribution that included SiC along with other materials (SiO_2_, graphite and Si). Furthermore, the variations in color center concentrations within the SiC crystals, coupled with the actual offsets of the pyrolysis positions, provide innovative insights and methodologies for enhancing the anti-counterfeiting capabilities of PUFs. Additionally, the integration of the MMPUF tags with the products enables applications in diverse sectors including microfluidic chips, bank cards, and medicinal tablets.

The first level of the MMPUF is dependent on the specific Raman spectra associated with the carbon-silicon bonds in SiC, which exhibited two characteristic peaks at 790 cm^−1^ and 960 cm^−1^, as shown in Figure 1b. By analyzing the Raman scattering signals, we could distinguish SiC from the other products, obtaining a random distribution of SiC in terms of spatial position and concentration, as depicted in Figure 1c. This level’s CRP was constructed from the 532 nm laser excitation and the corresponding output intensities of the Raman scattering spectra. Furthermore, during the formation of SiC nanocrystals, the specific locations and timings of the breakage and recombination of the polymer backbone and side chain groups in the substrate are completely random, resulting in a high degree of randomness in the final concentration and distribution of SiC. Simultaneously, since Raman spectroscopy analyzes molecular vibrations specific to certain bonds [33], it reflects the intrinsic properties of materials at the molecular level, exhibiting exceptional stability. Therefore, this strategy provides a secure and stable encoding method for anti-counterfeiting applications.

The second level of the MMPUF is based on the photoluminescence (PL) characteristics of V_si_ centers in SiC, as shown in Figure 1d. During the formation of SiC nanocrystals, the high-energy and non-ideal environmental conditions can lead to the creation of vacancy defects within some of the SiC crystals. Following thermal annealing, these vacancy defects capture surrounding free electrons, ultimately resulting in the formation of vacancy centers, as shown in Figure 1e. The different concentrations of color centers in SiC corresponded to different intensities of PL, which were positively correlated. By applying a 730 nm laser as the input challenge, we obtained the PL intensity distribution of the V_Si_ centers, constructing the corresponding CRP for the secondary anti-counterfeiting ES. Furthermore, the zero-phonon line (ZPL) of the V_si_ center was centered within the range of 1038 to 1133 nm, which falls within the infrared spectrum and is not visible to the naked eye. This characteristic enhances the level of encryption and protects the encoded information, facilitating a more secure and stable encoding method.

The third and fourth levels of the MMPUF rely on the offsets generated by the actual pyrolysis positions during LDW, relative to a reference position. These offsets can be represented in a two-dimensional spatial distribution as the offsets in the *X*-axis (Figure 1f) and *Y*-axis (Figure 1h) directions. In the process of LDW on samples arranged in an array, the specific offsets of each actual pyrolysis position relative to the reference position in the *X*-axis direction (Figure 1g) and *Y*-axis direction (Figure 1i) serve as the encoding sources for the third and fourth levels, respectively. The input challenge is a specific pyrolysis point, while the output response is the point offset along the *X*-axis and *Y*-axis, forming the CRP for the third and fourth levels. These offsets in the pyrolysis positions arise from the non-uniform random distribution of the energy absorption and conduction processes at the material surface during manufacturing, leading to randomness and inhomogeneity in laser energy deposition. When certain localized areas exhibit a faster increase in temperature compared with others, the high-temperature regions preferentially undergo pyrolysis, resulting in randomness in the pyrolysis positions. Moreover, this randomness is influenced by multiple complex factors including the uneven distribution of laser energy (non-uniform heat distribution and random shifts of the laser spot), material properties (surface morphology, thermal conductivity, and inhomogeneous material distribution), and uncertainties in the translational movements of the experimental equipment platform. The combined effects of these factors determine the propagation speed and distribution of heat within the material. Consequently, this ES possesses significant randomness, making it suitable for secure and stable encoding.

The anti-counterfeiting ES described across the four levels was based on four distinct physical properties involved in the preparation process of V_si_ centers. Raman scattering is a physical property related to the C-Si and is not connected to the defect luminescence or pyrolysis offsets in SiC. Thus, the first-level anti-counterfeiting strategy is completely independent of the subsequent three levels. The PL of V_si_ is a quantum property of SiC, which is unrelated to the offsets of the actual pyrolysis positions during fabrication. Therefore, the encoding of the second level of the PUF is also independent of the third and fourth level encodings. The offsets that occur in the *X*- and *Y*-axes due to pyrolysis position shifts are influenced by the laser parameters and substrate characteristics, but the factors affecting these offsets in the *X*-axis and *Y*-axis are necessarily uncorrelated. Additionally, since the *X*- and *Y*-axes are orthogonal in space, the third-level ES is independent of the fourth level. The introduction of this multilevel multimodal ES enhances the effectiveness of anti-counterfeiting encryption and significantly improves the EC of the PUF.

### 3.2. Fabrication and Characterization of MMPUF

**Figure 2:**
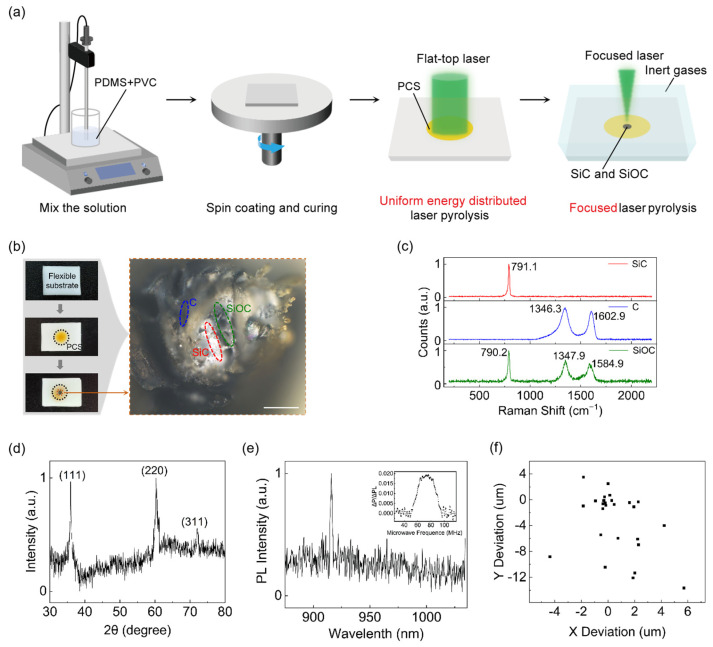
Fabrication and characterization of the V_Si_ center PUF based on LDW. (**a**) Schematic diagram of the fabrication process for the MMPUF. (**b**) Manufacturing process of an individual sample in the PUF and the optical image of the product (scale bar: 100 μm). (**c**) Raman spectrum of the prepared samples. (**d**) X-ray diffraction (XRD) analysis of the fabricated SiC. (**e**) PL and ODMR spectra of the V_Si_ centers in the MMPUF. (**f**) Scatter plot of the offsets in the *X*-axis and *Y*-axis directions of the actual pyrolysis positions relative to the reference point.

This work developed a manufacturing process for V_Si_ centers through LDW. The manufacturing process includes the following steps: (1) preparation of a mixed solution; (2) spin coating and thermal annealing for curing; (3) plateau laser pyrolysis; and (4) Gaussian laser pyrolysis in an inert gas environment, as shown in Figure 2a. Initially, a flexible sheet material was formed using PDMS and PVC as the base materials, followed by the first pyrolysis of the flexible substrate using a flat-top laser, resulting in the formation of SiC precursor polycarbosilane (PCS) and PCS-carbon (PCS-C) regions. Subsequently, a focused Gaussian laser beam was applied for secondary pyrolysis on the precursor region, leading to the formation of SiC crystals and color centers, as shown on the left side of Figure 2b. The right side of Figure 2b presents the distribution map of the products obtained under optical microscopy. According to the Raman scattering spectrum shown in Figure 2c, the resulting products mainly consisted of SiC, carbon (C), and silicon oxycarbide (SiOC), which corresponded to Raman peaks at 791.1 cm^−1^, 1346.3 cm^−1^ and 1602.9 cm^−1^ as well as peaks at 790.2 cm^−1^, 1347.9 cm^−1^, and 1584.9 cm^−1^, respectively.

To further elucidate the composition and crystal structure of the material, XRD analysis was performed. As depicted in Figure 2d, three distinct diffraction peaks were observed at 2θ = 35.91°, 60.31°, and 71.98°, corresponding to the (111), (220), and (311) crystallographic orientations of the SiC nanocrystals, respectively. Figure 2e displays the PL spectrum and the ODMR signal spectrum of the V_Si_ centers. The PL spectrum exhibited a peak at 915 nm, while the ODMR spectrum revealed a prominent peak near 72 MHz. The minor peaks present in the ODMR signal were attributed to small, inhomogeneous magnetic fields arising from environmental and instrumental effects during the measurements [34]. Figure 2f presents a scatter plot that depicts the spatial distribution of offsets in the actual pyrolysis positions relative to a reference point, intuitively demonstrating the random uncertainty in the offsets along the *X*- and *Y*-axes.

### 3.3. Encoding of MMPUF

The core of the digitization process for the MMPUF is binarization. The selection of the threshold during the binarization encoding is crucial, as it directly impacts the EC of the PUF. In this work, we employed the principle of maximizing entropy to determine the optimal threshold that yields the maximum amount of information. Values greater than the threshold are defined as 1, while values less than the threshold are defined as 0. When the counts of 0 s and 1 s are equal, with each occupying 50% of the total, the maximum number of random combinations of the binary code is achieved, resulting in the highest information entropy and optimal EC for the PUF [35].

**Figure 3:**
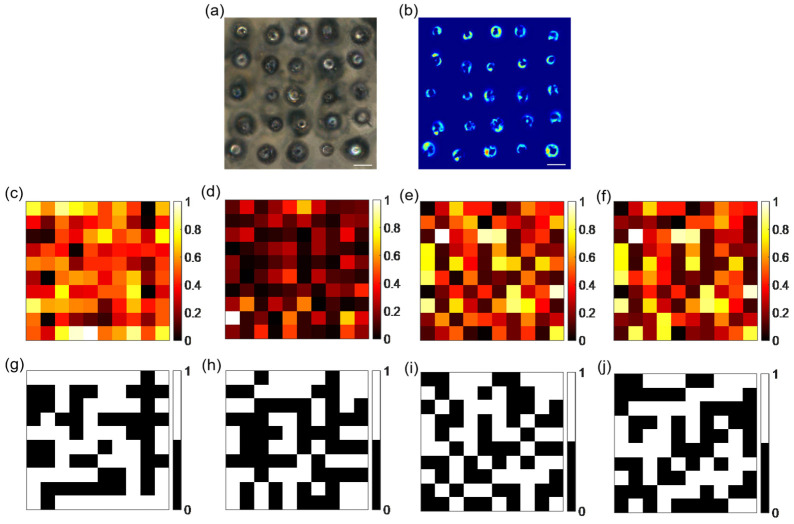
Encoding of MMPUF. Optical (**a**) and fluorescence (**b**) maps of multilevel multimodal PUF (scale bar 30 μm). Mapped encoded thermal maps (**c**,**d**) based on Raman, PL, and laser pyrolysis positions at the *x*- and *y*-axis offsets and corresponding binarization maps (**g**–**j**) for each level of encoding.

The first level of encoding employs laser as the input challenge for the PUF, with the output presented in the form of Raman signals. By extracting the Raman signal intensity from each point of the label and merging the images into a 10 × 10 pixel grid, we obtained the Raman mapping heat map (Figure 3c) of the PUF label along with its corresponding binary encoding map (Figure 3g). This process successfully encodes the Raman signal intensities into digital keys.

The second level of encoding employs excitation laser as the input challenge for the PUF label. Fluorescence images are captured and processed to produce grayscale images, from which the grayscale values of each pixel are extracted. Figure 3a,b shows the optical and fluorescence images, respectively. The image pixels are then merged into a grid of 10 × 10 pixels, resulting in a mapping image (Figure 3d) of the PL intensity and its corresponding binarized map (Figure 3h). This process effectively encodes the PL signals into digital keys.

The third level of encoding relies on the offsets that occur along the *X*-axis during laser processing pyrolysis. Each instance of laser pyrolysis is influenced by a combination of factors related to the laser and the material, resulting in varying offsets. The output along the *X*-axis manifests as different offsets, generating an offset mapping image (Figure 3e) of the offsets along the *X*-axis and their corresponding binary encoding image (Figure 3i). The conversion of the offsets in the *X*-axis direction of the actual pyrolysis position into the encoding of a digital key is realized.

The fourth level of encoding is derived from the offsets along the *Y*-axis generated during the laser processing pyrolysis. Different pyrolysis positions, under the influence of the applied challenge, produce various offsets in the *Y*-axis direction. The mapping image (Figure 3f) of the offset matrix along the *Y*-axis and the corresponding binary coding matrix map were obtained (Figure 3j). This effectively encodes the offsets in the actual pyrolysis positions along the *Y*-axis into digital keys.

The MMPUF employs a multilevel encoding architecture where each layer operates on a 10 × 10 pixel grid. Through the binarization of physical responses, each pixel generates a 1-bit binary output, resulting in a 100-bit cryptographic key per layer (10 × 10 pixels). By integrating four independent encoding layers, the system synthesizes a 400-bit composite key (4 × 100 bits). Meanwhile, the binary encoding scheme assigns each pixel two responses (0 or 1), with each layer containing 100 pixels. Consequently, the four independent encoding layers achieve a high encoding capacity of 2^4×100^.

### 3.4. Performance of MMPUF

By analyzing the performance metrics of the multilevel multimodal PUF, we can assess its security and application potential. The performance evaluation of PUF tags primarily includes repeatability, uniqueness, and spatial autocorrelation.

**Figure 4:**
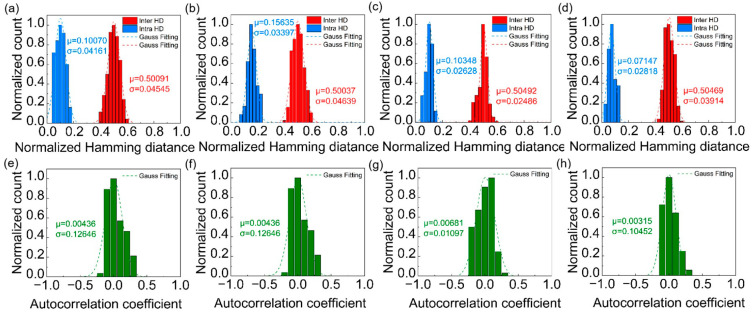
Performance of MMPUF encoding. Inter-HD and intra-HD based on (**a**) Raman encoding, (**b**) PL encoding, (**c**) *X*-axis offsets, and (**d**) *Y*-axis offsets. Autocorrelation functions for each encoding level are shown in (**e**–**h**).

Repeatability measures the consistency of a PUF’s responses under different environmental conditions and repeated trials. A robust PUF should maintain stable responses over time and across various operational environments. Specifically, for a given PUF tag, the output response should remain consistent when the same challenge is input multiple times. The intra-Hamming distance (intra-HD) can be used to evaluate the degree of variation between the two responses generated by applying the same challenge to the same PUF twice. It represents the repeatability of the PUF in generating random keys. In an ideal scenario, the intra-HD would be 0. By calculating the intra-HD for 50 coding images from the same PUF for each level of encoding, we obtained the intra-HDs for the MMPUF at various encoding layers, as shown in the blue graphs of Figure 4a–d. The average intra-HDs at each encoding level were 0.10070, 0.15635, 0.10348, and 0.07147, respectively, indicating a high reproducibility of the binary keys across all encoding layers. Additionally, we measured the fluorescence signals of the MMPUF tag across a temperature range of −30 °C to 70 °C with a step size of 1 °C and a humidity range of 30% to 80% with a step size of 1%. The results indicate that fluorescence intensity variations are independent of temperature and humidity and remained below 0.37%, which can be attributed to environmental noise. This further validates the stability and reliability of the MMPUF.

Uniqueness is an important metric for assessing whether a PUF tag can be distinguished from other PUF tags, and is typically quantified using the inter-Hamming distance (inter-HD). Specifically, applying a particular challenge to two different PUF tags allowed us to evaluate the difference between the outputs of the two responses using the inter-HD. By utilizing 200 coding images from 50 different PUFs, we analyzed the uniqueness of the MMPUF across various encoding layers, with results presented in the red bar graphs of Figure 4a–d. The average inter-HDs obtained for each encoding layer were 0.50091, 0.50037, 0.50493, and 0.50496, respectively. These results indicate that the binary keys generated at each encoding layer exhibit excellent uniqueness, providing strong evidence of the significant capability of the MMPUF in generating unique identifiers. This further validates its distinctiveness and unclonability. Additionally, we calculated the mean spatial autocorrelation coefficients for the various encoding layers of the MMPUF, which were 0.00436, 0.07319, 0.00681, and 0.00315, respectively. These values confirm that the multilevel multimodal PUF demonstrates low autocorrelation, thereby exhibiting higher security against correlation attacks.

### 3.5. Applications of MMPUF

**Figure 5:**
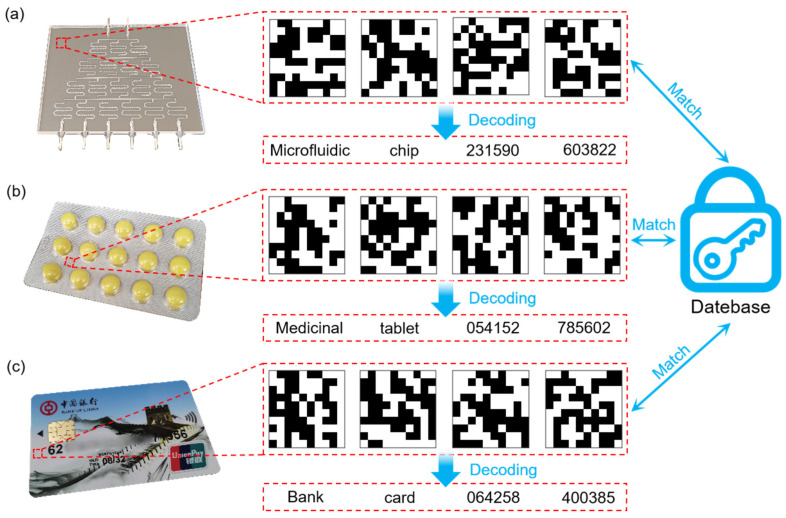
Applications of MMPUF encoding. Demonstration of authentication utilizing MMPUF in (**a**) microfluidic chips, (**b**) drugs, and (**c**) bank cards.

The MMPUF tags developed in this study can be applied in products such as microfluidic chips containing silicon sources (Figure 5a) and medicinal tablets containing carbon sources (Figure 5b) as well as bank cards (Figure 5c). Using the self-built database system that was developed by our team, combined with a multilevel encoding scheme, various challenges were input to generate corresponding responses, thereby forming a mapping relationship between the encoded information and its plaintext [30]. Additionally, our self-developed QR code system was utilized for information encryption and decryption. The authentication process involved extracting the four-level encoding and matching it with the database to ultimately retrieve the complete plaintext.

The MMPUF not only possesses the advantages of traditional multilevel PUF tags, but also benefits from its flexible substrate, allowing for applications in a wider range of fields. Furthermore, the developed PUF tags utilize a doping manufacturing process, incorporating missing elements into product materials that contain silicon-based or carbon-based components for in situ fabrication. The LDW technology used ensures high precision with minimal damage. The size of a single pyrolysis point is about 30 μm, and the entire MMPUF tag with the pyrolysis point array is within 200 μm. This ensures that the original performance of the product remains almost unchanged. This integration of the PUF tags into the items means that they are no longer simply attached to the surface. Instead, they are directly embedded within the items, becoming an inherent part of them. This approach eliminates the risks associated with traditional external tags, which are easily removable or subject to tampering, significantly enhancing the security and robustness of anti-counterfeiting measures.

## 4. Conclusions

In summary, we proposed a MMPUF based on LDW to manufacture V_Si_ centers, exploring not only the general properties of the materials themselves, but also their fluorescence characteristics and the pyrolysis offset features generated during the fabrication process, which provides a novel encoding solution for improving the PUF encoding capacity. A four-level independent ES was constructed based on the spatial random distribution of the SiC and V_si_ concentrations as well as the offsets of the laser pyrolysis position on the *X*- and *Y*-axes. This multilevel multimodal ES enables exponential growth in PUF EC at a low cost and allows for simple and rapid authentication through a self-built database system. The stability and unclonability of the MMPUF were validated by calculating the HDs and spatial autocorrelation coefficients. Furthermore, the high precision and small-scale nature of LDW was combined with the doping manufacturing process, which enabled label-product integration and significantly enhanced the stability and tamper resistance of the MMPUF. The applicability of this approach for authentication in microfluidic chips, medicinal tablets, and bank cards was demonstrated. Therefore, the MMPUF proposed in this study significantly improves system security and reliability, making it a promising solution for combating counterfeit products and is applicable in various anti-counterfeiting fields.

## Data Availability

The original contributions presented in this study are included in the article. Further inquiries can be directed to the corresponding author.
